# Polysaccharides from *Agaricus bisporus *and *Agaricus brasiliensis *show similarities in their structures and their immunomodulatory effects on human monocytic THP-1 cells

**DOI:** 10.1186/1472-6882-11-58

**Published:** 2011-07-25

**Authors:** Fhernanda R Smiderle, Andrea C Ruthes, Jeroen van Arkel, Wasaporn Chanput, Marcello Iacomini, Harry J Wichers, Leo JLD Van Griensven

**Affiliations:** 1 Plant Research International, Wageningen University and Research, Bornsesteeg 1, 6708 PD Wageningen, The Netherlands; 2 Departamento de Bioquimica e Biologia Molecular, Universidade Federal do Paraná, Centro Politécnico, CP 19046, Curitiba-PR, Brasil; 3 Wageningen University and Research Centre, Bornse Weilanden 9, 6708 WG Wageningen, The Netherlands

**Keywords:** *Agaricus bisporus*, *A. brasiliensis*, α-glucan, β-glucan, mannogalactan, immunomodulation

## Abstract

**Background:**

Mushroom polysaccharides have traditionally been used for the prevention and treatment of a multitude of disorders like infectious illnesses, cancers and various autoimmune diseases. Crude mushroom extracts have been tested without detailed chemical analyses of its polysaccharide content. For the present study we decided to chemically determine the carbohydrate composition of semi-purified extracts from 2 closely related and well known basidiomycete species, i.e. *Agaricus bisporus *and *A. brasiliensis *and to study their effects on the innate immune system, in particular on the *in vitro *induction of pro-inflammatory cytokines, using THP-1 cells.

**Methods:**

Mushroom polysaccharide extracts were prepared by hot water extraction and precipitation with ethanol. Their composition was analyzed by GC-MS and NMR spectroscopy. PMA activated THP-1 cells were treated with the extracts under different conditions and the production of pro-inflammatory cytokines was evaluated by qPCR.

**Results:**

Semi-purified polysaccharide extracts of *A. bisporus *and *A. brasiliensis *(= *blazei*) were found to contain (1→6),(1→4)-linked α-glucan, (1→6)-linked β-glucan, and mannogalactan. Their proportions were determined by integration of ^1^H-NMR signs, and were considerably different for the two species. *A. brasiliensis *showed a higher content of β-glucan, while *A. bisporus *presented mannogalactan as its main polysaccharide. The extracts induced a comparable increase of transcription of the pro-inflammatory cytokine genes IL-1β and TNF-α as well as of COX-2 in PMA differentiated THP-1 cells. Pro-inflammatory effects of bacterial LPS in this assay could be reduced significantly by the simultaneous addition of *A. brasiliensis *extract.

**Conclusions:**

The polysaccharide preparations from the closely related species *A. bisporus *and *A. brasiliensis *show major differences in composition: *A. bisporus *shows high mannogalactan content whereas *A. brasiliensis *has mostly β-glucan. Semi-purified polysaccharide extracts from both *Agaricus *species stimulated the production of pro-inflammatory cytokines and enzymes, while the polysaccharide extract of *A. brasiliensis *reduced synthesis of these cytokines induced by LPS, suggesting programmable immunomodulation.

## Background

Mushroom polysaccharides have traditionally been used for the prevention and treatment of a multitude of disorders like infectious illnesses, cancers and various autoimmune diseases. Bioactive polysaccharides are recognized by membrane receptors in leukocytes and macrophages, leading to proliferation and differentiation of immune cells [1,2]. These activities are responsible for enhancing the innate and cell-mediated immune responses, and consequently, for the induction of antitumoral and bactericidal effects [3,4].

Dendritic cells (DC's) and macrophages play important roles in many host reactions. They process antigen material and present it on their surface to other cells of the immune system. That is, they function as antigen-presenting cells to the T cells controlling immunity. Macrophages might behave both as pro-inflammatory cells to prevent e.g. infectious disease, as well as anti-inflammatory cells with a reparatory effect, as in wound healing [5]. By binding to their receptors, the bioactive polysaccharides activate various immune pathways like phagocytosis, complement activity, and respiratory burst and also the production of cytokines such as tumor necrosis factor-α (TNF-α), different kinds of interleukins (IL's) and enzymes as cyclooxygenase-2 (COX-2) [6,7]. All these effects collaborate to modulate cell differentiation and proliferation, enabling the host to defend itself against pathogens and tumors.

The monocytic THP-1 human myeloid leukemia cell line [8] expresses the Fc receptor and can be induced by phorbol 12-myristate 13-acetate (PMA) to differentiate into macrophage-like morphology [9]. Upon PMA treatment the suspension culture changes and cells become adherent to glass and plastics and show an increase in mitochondrial and lysosomal numbers and in cytoplasmic to nuclear ratio, as well as an altered differentiation dependent on cell surface markers in a pattern similar to monocyte-derived macrophages [10]. In addition they have a high absorption capacity for latex micro beads and express a cytokine profile that resembles macrophages.

*Ganoderma lucidum *polysaccharides have been found to induce monocytic THP-1 cells into dendritic cells [11] if added together with GM-CSF/IL-4, and recently it was published [12] that *G. lucidum *polysaccharides induced cytokine secretion and cell death in the same cell line. Also a heteromannan isolated from *Morchella esculenta *was able to activate the expression of NF-κB in THP-1 cells [13].

Among the polysaccharides that are encountered in mushrooms and have been tested *in vitro *and *in vivo *there are α-glucans, β-glucans, heterogalactans, heteroglucans, and others. The immunomodulatory effects are attributed mainly to (1→3)-β-glucans and (1→3)(1→6)-β-glucans [14,15]. Linear (1→6)-β-glucans and (1→4)-α-glucans of *A. brasiliensis *were shown to present antitumoral activities [16]. Interestingly, Ito et al. (1997) [17] have reported that macrophage activation and an alteration of complement factor C3 is necessary for the induction of an anti-tumor effect when *A. brasiliensis *polysaccharides are used.

Heteropolysaccharides isolated from different basidiomycetes were also considered as bioactive polymers, showing a large diversity of structures and effects. Galactoglucomannan (*L. edodes*), xyloglucan (*P. pulmonarius*), and glucogalactan (*G. lucidum*) are examples of these molecules [1].

The range of extractions and isolation procedures used nowadays [2,18] to separate carbohydrate contents from mushrooms, leads to different yields and compositions of bioactive materials. Usually the extracts obtained are not completely pure, retaining small amounts of proteins, phenolic components, and/or other carbohydrates that can also influence the biological effects. It was observed that proteins, terpenoids, steroids, and fatty acids all can function as immunomodulators, respectively as cytotoxic, antitumor, antibactericidal, and antihypertension compounds [1].

Since the different components of a mushroom extract may act in synergy, careful studies have to be carried out to compare the activity of isolated compounds with those of crude mushroom extracts and whether whole mushrooms provide benefits that go beyond those achievable with isolated constituents. It remains therefore necessary to perform chemical analyses of the crude extracts to evaluate their components, and to compare these biological effects with the activities observed for the isolated agents.

For the present study we decided to chemically determine the carbohydrate composition of semi-purified extracts from 2 closely related and well known basidiomycete species, i.e. *Agaricus bisporus *and *A. brasiliensis *and to study their effects on the innate immune system, in particular on the *in vitro *induction of pro-inflammatory cytokines, using THP-1 cells.

## Methods

### Cell culture

The human monocytic cell line THP-1 (Cell Lines Service, Eppelheim, Germany) was grown in RPMI 1640 culture medium (Sigma, cat. R8758) supplemented with 10% heat-treated newborn calf serum Sterile A (Gibco, cat. 161010-159) and 100 U/mL resp. 100 ug/mL penicillin/streptomycin (P/S) (Sigma-Aldrich), at 37°C in 5% CO_2 _in a humidified incubator.

### Macrophage differentiation and stimulation

The mature macrophage-like state was induced by treating THP-1 monocytes (500,000 cells/mL) for 48 h with 30 ng/mL phorbol 12-myristate 13-acetate (PMA; Sigma) in 24-wells polystyrene tissue culture plates (Costar) with 1 mL cell suspension in each well. The medium was then removed and replaced by fresh medium containing the different test samples.

### Gene expression kinetics by Real-Time PCR

Total RNA was isolated by using RNeasy mini kit (Qiagen, USA) with a RNase-free DNase (Qiagen) treatment for 15 min according to the manufacturer's instructions. Complementary DNA (cDNA) was synthesized from isolated RNA with Taqman Reverse Transcription Reagent kit (Applied Biosystems, USA). Expression levels of each gene were measured in duplicate reactions, performed with the same cDNA pool, in the presence of the fluorescent dye (iQ SYBR Green Supermix) using an iCycler iQ instrument (Bio-Rad Laboratories). The experiments were performed in a 20 μL reaction volume with specific primer pairs [19], and the conditions of real-time quantitative PCR were as follows: denaturation at 95°C for 3 min and amplification by cycling 40 times at 95°C for 10 s and 60°C for 30 s. Glyceraldehyde-3-phosphate dehydrogenase (GAPDH) was chosen for normalisation. The PCR of all products were subjected to a melting curve analysis to verify the single amplification product. The relative messenger RNA (mRNA) expression were presented as described in Chanput et al. (2010) [19]: the values were expressed as fold change relative to the value at time point zero, calculated as ΔΔCt [ΔΔCt = 2^(Ct_GAPDH _- Ct_Sample_)] [20]. All experiments were performed with the same amount of cells (0.5 × 10^6 ^per ml) and the same quantity of RNA input. qPCR was performed twice on each sample, in duplicate.

### Mushroom extracts preparation

The crude extracts from fruiting bodies of *Agaricus bisporus *(J.E. Lange) Imbach strain Sylvan A15, which is a commercial substrain of Horst U1 (ATCC 62462) and *Agaricus brasiliensis *Wasser et al. (syn. *Agaricus blazei *Murill) strain M7700, obtained from Innerlife B.V. (Venlo, The Netherlands) were prepared by hot water extraction as described before [21] and concentrated to > 35° Brix for cold storage. Polysaccharides were semi-purified by repeated precipitation with two volumes of 96% ethanol. The precipitate was dissolved in water and then centrifuged at 12,000 rpm in an Eppendorf centrifuge to remove residual solids. The solution was three times frozen and thawed and the precipitate was centrifuged as before. The resulting pellet (HWP) and the soluble fraction (HWS) were lyophilized. Both fractions were tested on THP-1 cells.

### Analysis of monosaccharide composition by GC-MS

Each polysaccharide fraction (1 mg) was hydrolyzed with 2 M TFA at 100°C for 8 h, followed by evaporation to dryness. The dried carbohydrate samples were dissolved in 0.5 N NH_4_OH (100 μL), held at room temperature for 10-15 min in reinforced 4 ml Pyrex tubes with Teflon lined screw caps. NaBH_4 _(1 mg) was added, and the solution was maintained at 100°C for 10 min, in order to reduce aldoses to alditols [22]. The product was dried and excess NaBH_4 _was neutralized by the addition of acetic acid or 1 M TFA (100 μL), which was removed following the addition of methanol (×2) under a N_2 _stream in a fume hood. Acetylation of the Me-alditols was performed in pyridine-Ac_2_O (200 μl; 1:1, v/v), heated for 30 min at 100°C. The resulting alditol acetates were analyzed by GC-MS, and identified by their typical retention times and electron impact profiles. Gas liquid chromatography-mass spectrometry (GC-MS) was performed using a Varian (model 3300) gas chromatograph linked to a Finnigan Ion-Trap model 810 R-12 mass spectrometer, with He as carrier gas. A capillary column (30 m × 0.25 mm i.d.) of DB-225, held at 50°C during injection and then programmed at 40°C/min to 220°C or 210°C (constant temperature) was used for qualitative and quantitative analysis of alditol acetates and partially O-methylated alditol acetates, respectively [23].

### Analysis of monosaccharide composition by HPLC

The samples were hydrolyzed with 2 M TFA at 100°C overnight, followed by evaporation to dryness. The residual TFA was removed by two evaporation cycles with 0.5 mL of MeOH, and the final residue was dissolved in 0.5 mL of H_2_O.

After 100 fold dilution monosaccharides were determined using a Dionex HPLC system (Dionex Corp. Sunnyvale, Cal. USA) fitted with a Carbo Pac PA-1 column (4-250 mm), and a 25 μL sample loop with 20 mM NaOH isocratic solution (1 mL min-1) as the mobile phase. An ED40 electrochemical detector fitted with a pulsed amperometric cell was used. Glucose and galactose were used as standards.

### Spectroscopy analysis

Nuclear magnetic resonance (NMR) (^13^C and coupled ^1^H(obs.),^13^C heteronuclear single quantum correlation (HSQC) spectra were obtained using a 400 MHz Bruker model DRX Avance spectrometer incorporating Fourier transform, as described before in detail [24]. Samples were dissolved in D_2_O and examined at 70°C. Chemical shifts are expressed in ppm (δ) relative to the resonance of D_2_O at δ 30.2 (^13^C) and 2.22 (^1^H).

### Determination of the phenolic content of the extracts

Folin assay was used for the phenolic determination of the extracts [25]. 100 μL Folin's phenol reagent was added to each sample (50 μL) and the solutions were mixed well. Then, 400 μL Na_2_CO_3 _was added to each mixture. The absorption of 200 μL of each sample was measured at 590 nm in the SPECTRAFluor spectrophotometer. Gallic acid was used as a standard in concentrations ranging from 12.5 to 50 μg/mL.

### Statistical Analysis

The results are expressed as mean ± standard deviation of duplicate cultures of two representative experiments. Statistical significance was determined using one-way analysis of variance (ANOVA) followed by Bonferroni's test. P ≤ 0.05 was considered statistically significant. The graphs were drawn and the statistical analyses were performed using GraphPad Prism version 5.01 for Windows (GraphPad Software, San Diego, CA, USA).

## Results

### Chemical analysis of the extracts

The semi-purified polysaccharide extracts were analyzed for phenol content as well as for their monosaccharide composition. The phenol components were measured by the Folin-Ciocalteau method based on the chemical reduction of the reagent [25], while the monosaccharide composition was analysed by HPLC. *A. bisporus *(ABS) and *A. brasiliensis *(ABL) extracts contained 0.96 and 0.78 μg phenol per mg of dry matter, respectively. Wei et al. (2008) [26] had found 3.4% and 2.1% of phenol content in *A. bisporus *and *A. brasiliensis*, respectively. The latter authors observed that polysaccharides of *A. brasiliensis *and also a brown-colored polysaccharide/phenol complex of *A. bisporus *were very active ROS generators. It has been published that polysaccharides are able to bind to polyphenols by intermolecular interactions [27], changing the molecular conformation of carbohydrates; therefore their presence should not be ignored.

The main monosaccharides of the samples and their respective proportions are given in Table 1. The extract from *A. bisporus *presented mainly glucose, mannose, galactose, and methyl-galactose. The presence of methyl groups was confirmed by GC-MS.

**Table 1:** Monosaccharide composition of the extracts.

Monosaccharides (%)						
**Sample**	**Glucose**	**Mannose**	**Galactose**	Gal-Me*	**Fucose**	**Ribose**

ABS	51.4	6.8	33.9	3.0	1.6	3.3
ABSE	43.0	10.3	36.6	3.3	2.0	4.8
ABL	66.7	4.9	23.1	-	5.6	-

The samples were analyzed by HPLC. *Confirmed by GC-MS.

**Figure 1:**
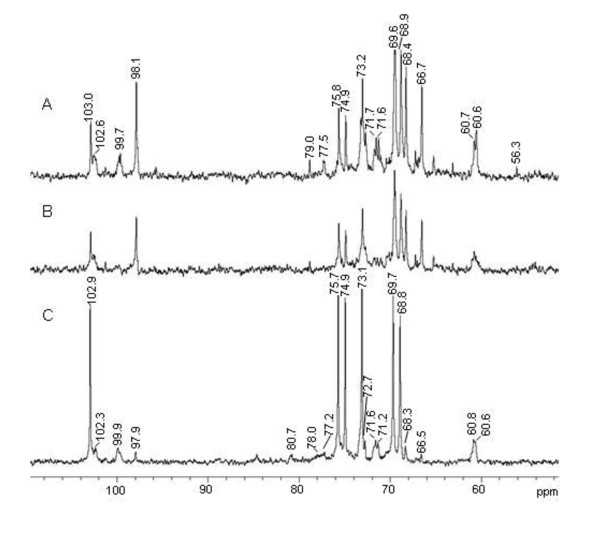
**^13^C-NMR spectra of *A. bisporus *extract, and *A. brasiliensis *extracts**. ^13^C-NMR spectra of *A. bisporus *extract (ABS) before (A) and after (B) α-amylase treatment (ABSE), and *A. blazei *extract (ABL) (C) in D_2_O at 50°C (chemical shifts are expressed in δ ppm).

**Figure 2:**
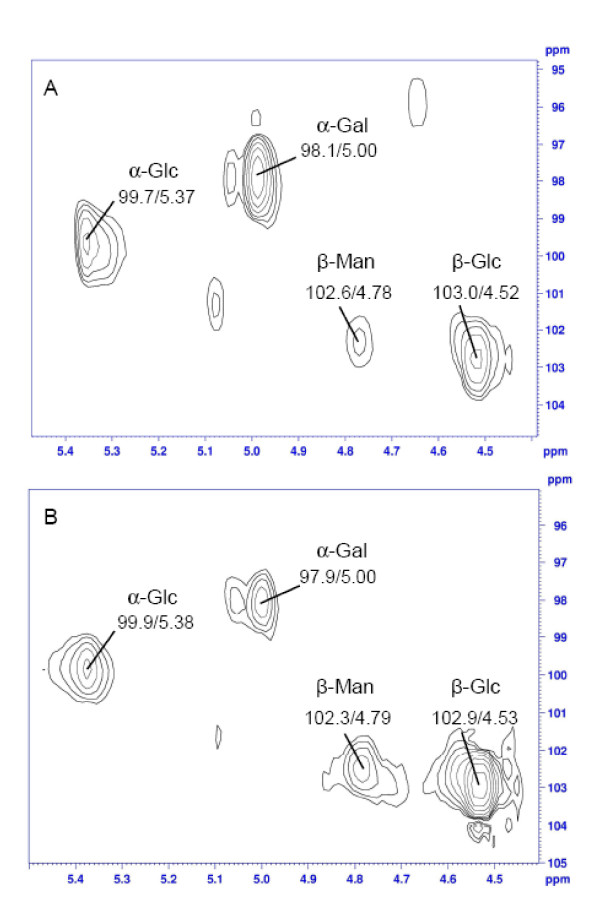
**Anomeric regions of HSQC spectra from *A. bisporus *extract (ABS) (A) and *A. blazei *extract (ABL) (B)**.

The ^13^C-NMR spectrum showed four signals in the anomeric region at δ 98.1, 99.7, 102.6, and 103.0 ppm (Figure 1a). The resolution and complexity of the signs indicates the presence of more than one polysaccharide in the sample. After treatment with α-amylase from *B. licheniformis *(250 U/mL) for 2 h at 50°C and re-precipitation with ethanol, the remainder (ABSE) showed a reduction of the level of glucose when analysed by GC-MS (Table 1). It can be seen in Figure 1b that the signs at δ 99.7 ppm and between δ 60.0 and 80.0 ppm do not appear in the spectrum anymore, showing clearly that there was a poorly branched α-glucan present with a main chain (1→4)-linked, which was degraded by the enzyme. The original signs at δ 98.1, 102.6, and 103.0 ppm were not reduced. The presence of an α-glucan in *A. bisporus *had already been described previously [24]. These authors showed by NMR experiments and methylation analysis that the α-glucan was composed of a (1→4)-linked main chain, substituted at O-6 by single units of α-glucose in the proportion 1:8. Normally, β-glucans are isolated from mushrooms [6]. We distinguished six signals in the ^13^C-NMR spectrum at δ 103.0; 75.8; 74.9; 73.2; 69.6; 68.4 ppm, typical from β-glucan (1→6)-linked, which was confirmed by the inversion of the sign at δ 68.4 in the DEPT experiment (data not shown). Several other low intensity signals were observed for this polysaccharide fraction and seem to be related to a polysaccharide composed mainly of galactose, as already observed for other basidiomycetes such as *Pleurotus ostreatus *[28], *P. pulmonarius *[29], *P. eryngii *and *P. ostreatoroseus *[30]. The latter authors isolated an α-galactan, while the previous ones described a mannogalactan from *Pleurotus *genus. In our study we found 10% of mannose in the ABS extract, and the spectra showed more complexity, giving a strong indication that a similar mannogalactan, as the one described by Jakovljevic et al. (1998) [28] and Smiderle et al. (2008) [29] is presented also in *A. bisporus*. Both authors isolated a polysaccharide with a main chain composed of (1→6)- α-galactose substituted at O-2 by β-mannose units. Interestingly, galactomannan and terminal mannose have been found to bind to mannose receptors in human phagocytic cells and exert immunomodulatory effects [13,31]. Furthermore, this heteropolymer contains natural methyl groups indicated by the sign at δ 56.3 ppm, and confirmed by GC-MS analysis showing the presence of 3-*O*-methyl-galactose. We decided to perform ^1^H-NMR and HSQC experiments to evaluate the proportion of each polysaccharide present in ABS extract. It was possible to integrate four main signals from the ^1^H-NMR spectrum (data not shown) based on HSQC anomeric region (Figure 2a), and determine the proportion of α-glucan (20.4%), β-glucan (23.7%), and mannogalactan (55.8%) in the extract (Table 2). These two samples partially purified polysaccharides (ABS) and the same after α-amylase treatment and re-precipitation (ABSE) were tested for their immuno activity properties in THP-1 cells.

**Table 2:** Proportions of polysaccharides in the extracts.

Polysaccharides	A. bisporus (%)	A. brasiliensis (%)
α-glucan	20.4	25.7
β-glucan	23.7	49.1
Mannogalactan	55.8	25.2

The polysaccharide fraction prepared of *A. brasiliensis *showed similar monosaccharide composition as the fraction observed for *A. bisporus *(Table 1), although the proportion of glucose was higher. The ^13^C-NMR spectrum (Figure 1c) also showed similarities with the other species regarding the presence of the signals, especially in the anomeric region. The high intensity signs arise from the linear (1→6)-β-glucan (δ 102.9; 75.7; 74.9; 73.1; 69.7; 68.8 ppm), as already described for *A. brasiliensis *[32]. The inversion of the sign at δ 68.8 ppm in the ^13^C-NMR DEPT spectrum proved the O-6 linkage (data not shown). Minor signs at δ 97.9 and 102.3 ppm confirmed the presence of the mannogalactan that we found for *A. bisporus*. Although some authors commented about the presence of galactose and mannose in *A. brasiliensis*, this heteropolymer was not well-characterized yet for these two species [16]. The (1→4)-linked α-glucan is present in higher amounts than in *A. bisporus*, even though the signal was not so evident at δ 99.9 ppm. The high concentration and solubility of the β-glucan evidences its signs in the spectrum, and reduces the resolution of the other molecules' signals. The α-glucan was also observed for *A. brasiliensis *and described by Gonzaga et al. (2005) [18]. By analyses of HSQC (Figure 2b) and integration of the areas in ^1^H-NMR spectrum (data not shown) it was possible to determine the proportion of each polysaccharide in the extract: α-glucan (25.7%), β-glucan (49.1%), and mannogalactan (25.2%) (Table 2). This extract was also tested on THP-1 cells. The protein content of both mushroom extracts was low since the NMR spectra showed no or few N-H signals (data not shown).

### Gene expression for inflammation-related cytokines and enzymes of THP-1 macrophages stimulated with extracts

**Figure 3:**
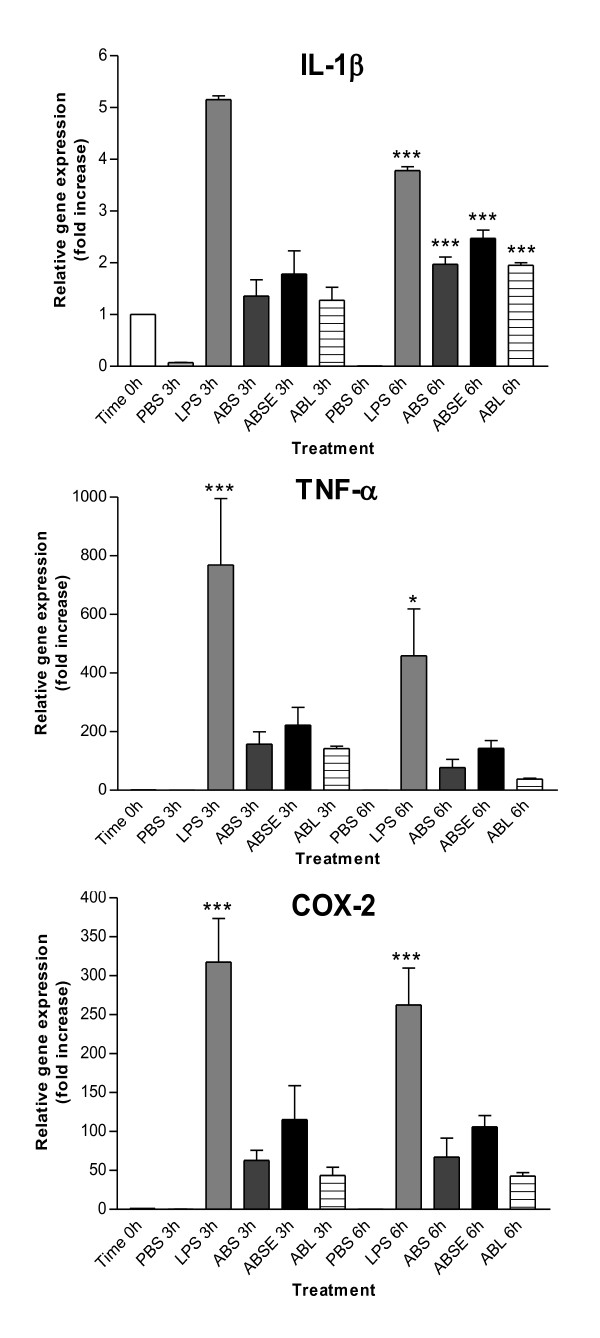
**mRNA expression level of genes for IL-1β, TNF-α, and COX-2 after treatment with mushroom extracts**. Negative control (PBS), positive control (LPS), *A. bisporus *extract (ABS), α-amylase treated *A. bisporus *extract (ABSE), and *A. blazei *extract (ABL). Statistical analyses were performed by means of one-way analysis of variance (ANOVA) followed by Bonferronis' test. The results represent the mean ± SD of duplicate cultures of two representative experiments. *p < 0.05; **p < 0.01; ***p < 0.001 versus negative control.

The extracts were added to THP-1 macrophages (after differentiation with PMA) at 250 μg/mL. Cells were harvested at the time points 0 h, 3 h, and 6 h and kept in lysis buffer at -20°C for the next step. The total RNA was isolated from the cells using RNeasy mini kit (Qiagen) and 1 μg was used to synthesize cDNA. The q-PCR analyses were pursued to evaluate the mRNA expression level of pro-inflammatory cytokine genes IL-1β and TNF-α and also the inflammation-related enzyme COX-2. Phosphate buffered saline (PBS; 50 μl) and lipopolysaccharide (LPS; 1 μg/ml) were used as negative and positive controls, respectively. It was possible to observe that both crude extracts from *A. bisporus *and *A. brasiliensis *induced the expression of TNF-α, IL-1β, and COX-2 (Figure 3). TNF-α showed a 150 fold increase of relative gene expression when compared to the time point 0 h. This result was observed for both mushrooms after 3 h of incubation. After 6 h the level of mRNA started to decrease, which was expected considering that this is the first cytokine to be produced under inflammatory stress [33,34]. IL-1β was expressed almost equally at both time points, ABS and ABL induced an almost 2-fold increase after 6 h treatment (p < 0.001). The extract treated with α-amylase showed higher stimulation compared to the others (2.5-fold increase; p < 0.001). The presence of the α-glucan interferes negatively with the activity; it probably blocks the active compounds or it interferes with their access to the membrane receptors, reducing cell stimulation and its effects.

Although the polysaccharides led to a high increase in production of TNF-α, and COX-2 mRNAs, the effect observed for the positive control LPS was still higher than the other samples.

**Figure 4:**
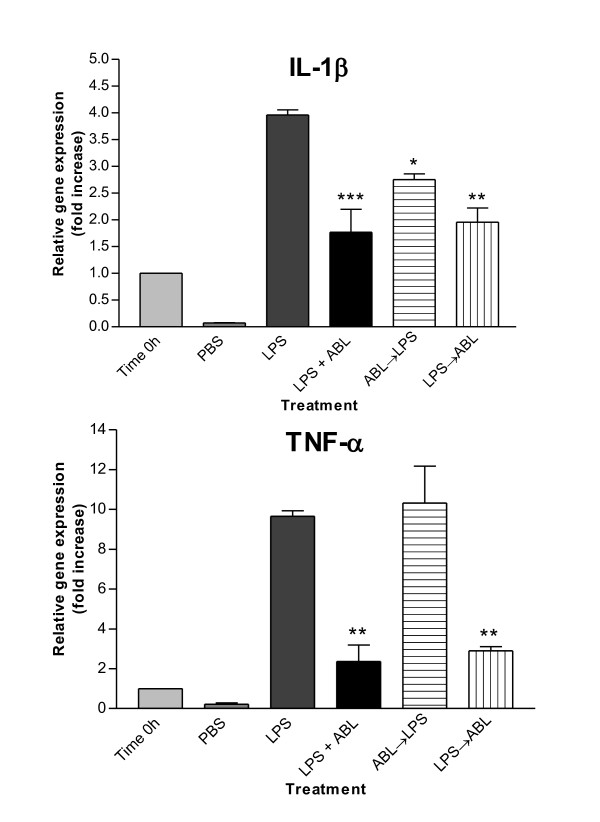
**mRNA expression level of genes for IL-1β, TNF-α after treatment with ABL**. Negative control (PBS) and positive control (LPS). Statistical analyses were performed by means of one-way analysis of variance (ANOVA) followed by Bonferronis' test. The results represent the mean ± SD of duplicate cultures of two representative experiments. *p < 0.05; **p < 0.01; ***p < 0.001 versus positive control.

The ideal situation would keep a balance among pro- and anti-inflammatory cytokines. To evaluate if the *A. brasiliensis *extract (ABL) was able to diminish the levels of TNF-α and IL-1β, we added the bacterial toxin LPS and ABL to the cells, in three different conditions, as described in table 3. The aim was to verify if the mushroom extract could possibly reduce the pro-inflammatory cytokines produced in the presence of LPS, or if it increases the effect of lipopolysaccharide. After 9 h with LPS, the cells showed 3.9 and 9.4 fold increase for IL-1β and TNF-α, respectively, compared to time 0 h (Figure 4). All conditions seemed to reduce the level of IL-1β induced by LPS stimulation, although the most effective was the simultaneous addition of ABL and LPS, which reduced the production of IL-1β in 56.5% (p < 0.001). While the most effective condition to modulate TNF-α was the simultaneous addition of both stimuli (reduction of 76.1%; p < 0.01) or the addition of ABL after 3 h with LPS (reduction of 69.8%; p < 0.01).

**Table 3:** Description of conditions for treating cells.

Condition	Time 0 h	After 3 h	After 6 h	After 9 h
Negative control	PBS	-	-	All cells were
Positive control	LPS	-	-	harvested
Stimulus at same time	LPS + ABL	-	-	
Prevention with extract	ABL	-	LPS	
Treatment with extract	LPS	ABL	-	

## Discussion

Basidiomycetes present different kinds of glucans and heteropolysaccharides. The common monosaccharide composition of these polymers is glucose, galactose, mannose, xylose, and fucose. Normally (1→3),(1→6)-β-glucans are extracted from these organisms, and also galactomannans, heteroglycans, and fucogalactans [2,4]. Mushrooms included in the same genera show more similarities in their composition, including the structure of carbohydrates [30]. In the present study, we compared two species from the genus *Agaricus*, which showed comparable NMR profiles. Both extracts contained mixtures of three main polysaccharides and their composition was comparable to what had been observed for other basidiomycete mushrooms, i.e. presenting glucose, galactose, mannose and fucose [16]. *A. brasiliensis *showed higher contents of β-glucan while *A. bisporus *presented mannogalactan as main polysaccharide. The proportion of each polysaccharide observed for both species varied significantly, and this may be an explanation for the differences encountered in the biological effects described by other authors. *A. brasiliensis *is known to be a medicinal mushroom and it has been widely used in Japan for many years for the treatment of cancer and other diseases [35]. *A. bisporus*, on the other hand, is mainly consumed as food; however there is quite some evidence concerning possible therapeutic properties as reduction of blood glucose [36] and cholesterol levels [37], generation of ROS species by human cells [26], and NO production stimulation [38].

In this study we evaluated the capacity of the mushroom extracts to stimulate the production of the pro-inflammatory cytokines TNF-α, IL-1β, and the enzyme COX-2 on THP-1 cells. In this assay, the samples were added to the cells and after different incubation periods they were harvested for analysis. The differences between both species were not significant. However a slight increase of expression was observed for the three transcripts induced by the α-amylase treated polysaccharide (ABSE) of *A. bisporus*. This treatment increased the content of β-glucan and mannogalactan, since it degrades only α-(1→4)-linkages. Although this glycogen-like molecule showed antitumor properties [16], there are no reports showing an immunostimulatory activity. Galactomannan from *M. esculenta *[13] and a polysaccharide from *G. lucidum*, containing glucose (58.1%), mannose (15.1%), and galactose (13.5%) had been found to stimulate THP-1 and showed an increase in NF-κB expression or were able to activate the differentiation to DC's, respectively [12]. Therefore, the presence of β-glucan may not be the only agent for the effects observed in the present study.

Macrophages contain specific membrane receptors that might bind polysaccharides and/or glycoproteins as Toll-like receptor 4 (TLR4), CD14, complement receptor 3 (CR3), scavenger receptor, dectin-1, and mannose receptor [4]. The binding to these receptors activates the transcription factor NF-κB, which controls the expression of multiple genes in activated monocytes and macrophages. Some of the genes regulated by NF-κB are the pro-inflammatory cytokines, chemokines, and inflammatory enzymes [13,39]. This could explain the induction of TNF-α, IL-1β, and COX-2 by THP-1 cells after the treatment with the extracts. It is known that the structure of the polysaccharides, as well as their conformation, molecular weight, and solubility in water may influence the receptor ligand interaction [2]. Moradali et al. (2007) [1] mentioned that triple-helix conformation of glucans and the presence of hydrophilic groups on the outside surface of the helix are important for their biological effect. Considering that the receptors are not specific for glucose polymers, it may be possible that the mannogalactan present in the extracts also bind the receptors, activating the nuclear transcription factor. Besides, the presence of the mannogalactan can also provide a well-suited conformation for the β-glucans, and facilitate their binding to dectin-1, the well-known receptor for glucans [39]. Further experiments should be performed to elucidate the mechanism of the immunomodulatory effects of these polymers.

A complementary study was performed to evaluate the capacity of ABL extract to reduce the expression of pro-inflammatory cytokines. The treatment reduced the expression of IL-1β, and markedly reduced TNF-α production. The addition of ABL concomitantly with LPS or after 3 h was found to reduce or avoid the expression of TNF-α and IL-1β, while the reduction, by adding ABL before LPS, was not effective. It was shown before that CD14 and Toll-like receptor 4 (TLR4) present in macrophages are essential for LPS recognition and consequently for responsiveness to this bacterial endotoxin [40]. Considering that these receptors are probably bound by polysaccharides, it is possible that there is competition between LPS and glucans/mannogalactans when added concomitantly. These experiments led to the conclusion that the semi-purified polysaccharide extracts of *A. bisporus *and *A. brasiliensis *can stimulate the production of pro-inflammatory cytokines and enzymes in THP-1 cells, as well as reduce the response to LPS.

It is well known that TNF-α and IL-1β are pro-inflammatory cytokines; therefore they can induce inflammation, fever and tissue damage. Blocking these chemokines could help relieve symptoms of inflammatory processes as in rheumatoid arthritis inflammatory bowel disease and other autoimmune diseases. Contrarily, as TNF-α and IL-1β play an important role against invasive pathogens, their induction may be relevant to increase e.g. antimicrobial resistance [41]. Eventual optimal application will depend on specifications of specific target groups.

The presence of α-glucan in the *A. bisporus *extract reduced its activity, showing that the β-glucan and the mannogalactan are the major bioactive agents. In addition *A. brasiliensis *polysaccharide extract reduced the LPS induced synthesis of TNF-α and IL-1β. Both extracts (ABS and ABL) presented comparable effects even though they showed significant differences in the proportion of β-glucan and mannogalactan in their composition. While *A. bisporus *presents 55.8% of mannogalactan and only 23.7% of β-glucan, *A. brasiliensis *is composed of 25.2% of mannogalactan and 49.1% of β-glucan.

## Conclusions

The closely related species *A. bisporus *and *A. brasiliensis *show major differences in polysaccharide composition: *A. bisporus *shows high mannogalactan content whereas *A. brasiliensis *has mostly β-glucan. Semi-purified polysaccharide extracts from both *Agaricus *species stimulated the production of pro-inflammatory cytokines and enzymes, while the polysaccharide extract of *A. brasiliensis *reduced synthesis of these cytokines induced by LPS, suggesting programmable immunomodulation.

## Competing interests

The authors declare that they have no competing interests.

## Authors' contributions

FS carried out the chemical analyses and qPCR, as well as the experiments with cells, and drafted the manuscript. AR participated on NMR experiments. JA carried out the HPLC analyses. WC participated in the design of the study with cells and the qPCR experiments. HW conceived of the study with cells and helped with discussion of results. MI contributed with the discussion of chemical results and the design of chemical procedures. LG carried out the polysaccharide extractions, and coordinated the draft of the manuscript, helping with the discussion of biological results and English correction of the text. All authors read and approved the final manuscript.

## Pre-publication history

The pre-publication history for this paper can be accessed here:

http://www.biomedcentral.com/1472-6882/11/58/prepub
